# Comparative evaluation of remineralisation potential using BioHAP, CPP-ACP and Enamelon on root dentin: An *in vitro*study

**DOI:** 10.6026/973206300221339

**Published:** 2026-03-31

**Authors:** Rashmi Patil, Rinky Hajong, Aslam Tariq Mohammed, Sruthi Keerthi, Pranoti Lavekar, Dimple Rakhecha, Rubeena Naaz, Md Kafeel Ahmed

**Affiliations:** 1 Department of Conservative and Endodontics, P.M.N.M Dental College and Hospital, Bagalkot, Karnataka, India; 2 Department of Conservative Dentistry and Endodontics, Government Dental College and Hospital, Silchar, Assam, India; 3 Department of Orthodontics, Narayana Dental College, Nellore, Andhra Pradesh, India; 4 Department of Pedodontics and Preventive Dentistry, Consultant in Hyderabad, Telangana, India; 5 Department of Public Health Dentistry, Private Practitioner, Pune, Maharashtra, India; 6 Department of Conservative Dentistry and Endodontics, Government Dental College and Hospital, Ahmedabad, Gujarat, India; 7 Private Practitioner, Dental Surgeon, SRK Dental Clinic, Hyderabad, Telangana, India; 8 Department of Periodontology and Implantology, MNR Dental College and Hospital, Sangareddy, Hyderabad, Telangana, India

**Keywords:** Root dentin, remineralisation, biomimetic hydroxyapatite (BioHAP), casein phosphopeptide-amorphous calcium phosphate (CPP-ACP), enamelon

## Abstract

Root caries and cervical dentin hypersensitivity affect older adults with gingival recession, requiring effective remineralisation strategies for root dentin preservation. Therefore, it is of interest to compare remineralisation potential of BioHAP, CPP-ACP and Enamelon on artificially demineralised root dentin from 60 extracted premolars using a 28-day pH cycling protocol. All agents significantly increased microhardness versus control (p<0.001), with BioHAP achieving superior recovery at 68.42±7.83% compared to other groups. SEM analysis confirmed BioHAP's enhanced mineral deposition and dentinal tubular occlusion, demonstrating superior structural repair capabilities. These findings advance restorative dentistry by establishing BioHAP as the optimal commercial agent for root dentin remineralisation and hypersensitivity management.

## Background:

The development of root caries and dentin hypersensitivity has become a common oral diseases especially in the aged populations exposed to gingival recession, leading to the exposure of roots. Structural and composition peculiarities of root dentin, such as increased organic content and reduced mineral density in relation to enamel, make root dentin more vulnerable to demineralisation and carious attack [1]. The rise in life expectancy around the world and retention of more teeth in elderly people has led to a higher occurrence of root surface lesions, which have necessitated the creation and testing of effective preventive and curative methods [2]. Root dentin contains almost 70/20/10 parts of inorganic minerals, hydroxyapatite crystals, organic matter and water, respectively. The existence of the dentinal tubules that are direct communication routes between the oral environment and the pulp of the teeth is what adds to the sensitivity of the exposed root surfaces, in addition to the possible routes of bacterial entry [3]. Knowledge of these structural peculiarities is critical in establishing specific remineralisation strategies that can be effective in recovering mineral content by covering the dentinal tubules. To achieve a demineralisation-remineralisation balance at the tooth surface, many factors such as salivary composition, dietary habits, oral hygiene practices and remineralising agent presence have a role to play. When the balance is shifted toward the demineralisation, mineral losses take place and incipient lesions are formed and can develop into cavitated caries if not treated [4]. Remineralisation, on the other hand, entails the process of depositing calcium, phosphate and other mineral ions in the demineralised tooth structure, which may reverse early lesions and restore mechanical properties.

Fluoride has long been regarded as the gold standard when it comes to caries prevention and remineralisation treatment. Nevertheless, the major mechanism of action of fluoride is the increase of calcium and phosphate ions precipitation by the saliva and a decrease in the rate of demineralisation, instead of the actual supply of mineral substrate [5]. This disadvantage has initiated studies on other and complementary remineralising agents that have the capability of providing bioavailable calcium and phosphate ions directly to the tooth surface. Casein phosphopeptide-amorphous calcium phosphate (CPP-ACP) is one of the established milk protein-derived remineralising technologies. The casein phosphopeptides stabilise calcium and phosphate ions in amorphous and bioavailable forms, which allows the diffusion into the subsurface lesions and later precipitation as hydroxyapatite [6]. Far less research has been conducted on the effectiveness of CPP-ACP in enamel remineralisation, but relative research has been conducted on the effect of CPP-ACP on root dentin [7]. Biomimetic hydroxyapatite (BioHAP) has been of particular interest as a fluoride-free remineralising agent that resembles the natural mineral part of dental hard tissues. Synthetic nanohydroxyapatite particles are highly biocompatible and capable of integrating into the existing tooth structure, possibly forming a biomimetic layer of apatite that blocks dentinal tubules and restores surface integrity [8]. Recent studies have also suggested the remineralising potential of BioHAP on enamel surfaces, with some encouraging results being reported on the possible dentin benefits [9]. Enamelon, which is a stabilised stannous fluoride and calcium sodium phosphosilicate (NovaMin), is a new technology that involves using fluoride technology and bioactive particles of glass. Calcium sodium phosphosilicate component releases calcium, sodium and phosphate ions when exposed to aqueous conditions, thus making it possible to stimulate mineral deposition with the stannous fluoride serving as an antimicrobial and anticaries action [10]. These synergistic actions of these components can provide better remineralisation than standard fluoride preparations. Modern studies have paid more attention to the comparison of different remineralising agents in controlled conditions to create evidence-based guidelines on clinical practice. The comparative effectiveness of various remineralising technologies on enamel has been assessed by a systematic review, which has shown a range of effectiveness of various forms of the product depending on the experimental conditions and specific formulation used [11]. Nonetheless, comparisons of BioHAP, CPP-ACP and Enamelon on root dentin surfaces per se are only found in a few studies in the literature [12]. The pH cycling model has proven to be popular in the *in vitro*remineralisation research due to its ability to mimic the dynamic nature of the oral cavity with alternating waves of demineralisation and remineralisation. This is a methodology that gives standardised conditions in the comparison of effects of treatments, as well as taking into consideration the cyclical nature of mineral exchange that happens *iin vivo* [13]. The outcome measures, such as surface microhardness, mineral content analysis and morphological assessment, give a full assessment of remineralisation effectiveness. Surface microhardness testing provides a non-destructive technique that is reliable to quantify the changes in the mineral in dental hard tissues. The percentage surface microhardness recovery (%SMHR) is a validated parameter for measuring the extent of remineralisation and is therefore directly comparable to the treatment modalities [14]. The methods, such as scanning electron microscopy (SEM) and energy-dispersive X-ray spectroscopy (EDX), give qualitative and quantitative data about the surface morphology and elemental structure, respectively. Although the number of articles describing research on remineralising agents is increasing, there are still major gaps in knowledge concerning the relative efficacy of modern products in just root dentin. The composition and structure of root dentin are different from those of enamel and thus, specific studies are required as opposed to extrapolating on the outcomes of enamel research [15]. Moreover, laboratory results need to be confirmed by research with physiologically relevant procedures in order to draw any clinical conclusions. Therefore, it is of interest to conduct a comparative and relative assessment and evaluation of the remineralisation ability of BioHAP, CPP-ACP and Enamelon on artificially demineralised root dentin using surface microhardness analysis, scanning electron microscopy and energy-dispersive X-ray spectroscopy.

## Materials and Methods:

## Ethical approval and design of the study:

The following is an experimental study carried out *in vitro*(the Department of Conservative Dentistry and Endodontics, University Dental Research Centre) from March 2023 to November 2023.

## Sample size calculation:

The power analysis was used as a determinant of sample size based on initial data and past literature on micro hardness recovery after the remineralisation treatment. Given that the difference in microhardness recovery between groups would be 15% as the minimum clinically significant difference, a standard deviation of 10, an 80% power and 5% significance value, at least 13 specimens per group was required. To counter the chances of the loss of specimens during the course of processing, 15 specimens were assigned to each group and this resulted in a total sample population of 60 specimens.

## Preparation and selection of the specimen:

A total of 60 fresh human premolars, which were harvested by extracting and retrieved in 0.1% thymol solution at 4°C, were collected and stored till used, in the 0.1% thymol solution at 4°C. Extraction Teeth had been used within three months.

## Inclusion and exclusion criteria:

Inclusion criteria included: intact root surfaces; free of caries; cracks or restorations; no developmental defects or fluorosis; full root formation; and extraction within the last three months. Exclusion criteria were: the teeth had undergone prior endodontic therapy; already had an abrasion, erosion, or abfraction lesion; had undergone a bleaching procedure; and specimens with observable structural defects.

## Preparation of root dentin specimens:

The cementoenamel junction was cementoenamel decorated with a low-speed diamond saw under constant water irrigation. The third cervical root was cut off and taken away in a length of 4 mm. Wet silicon carbide abrasive paper (600-grit) was used to remove the cementum layer, which revealed the underlying root dentin surface. Specimens were placed in acrylic resin blocks, which were self-curing and the buccal root dentin surface was exposed. To get standardised flat surfaces, the exposed surfaces were successively polished with 800, 1200 and 2000-grit silicon carbide papers and finally with 0.3 µm alumina suspension. To eliminate debris, samples were washed in deionised water under the ultrasonic mode for 10 minutes.

## Baseline microhardness evaluation:

A Vickers microhardness tester was used to measure the baseline surface microhardness with a diameter indenter having a load of 50 grams and duration of 15 seconds. Five marks were placed on each surface of specimen 100 µm apart and average was taken as the baseline microhardness (VHN baseline).

## ADP (Artificial Demineralisation Protocol):

Artificial demineralisation of specimens was performed in an acidic buffer solution that consisted of 2.2 mM calcium chloride, 2.2 mM sodium phosphate and 50 mM acetic acid and adjusted to pH 4.5. Single specimens were subjected to 30 mL of the demineralising solution at 37°C of time, but the solution was replaced every 24 hours. After demineralisation, surface microhardness was again measured on the same protocol (VHNdemin). Specimens that experienced at least a 30 per cent decrease in surface microhardness were incorporated in the study. Specimens that did not satisfy this requirement were substituted.

## Randomisation and group allocation:

Demineralised samples were randomly divided into four groups (n=15 each) by use of computer-generated randomisation:

The following were the samples:

[1] Group A (BioHAP): Bio repair Plus toothpaste, with 20% zinc-substituted carbonate hydroxyapatite nanoparticles.

[2] Group B (CPP-ACP): GC Tooth Mousse, which has 10% casein phosphopeptide-amorphous calcium phosphate.

[3] Group C (Enamelon): Enamelon toothpaste that has stabilised stannous fluoride (1100 ppm) with calcium sodium phosphosilicate.

[4] Control: Artificial saliva alone (Group D).

## pH cycling and treatment protocol:

The 28-day pH cycling program was adopted to replicate the dynamic oral environment.

The daily cycle consisted of:

[1] Treatment use: The respective remineralising agents were put on the surface of the specimen to be treated during 3 minutes twice per day (mimicking the morning and evening brushing) and by using a microbrush applicator. Artificial saliva application was done on control specimens.

[2] Demineralisation stage: The specimens were placed into fresh demineralising solution (pH 4.5) for three days and incubated for 3 hours daily to mimic acid challenges.

[3] Remineralisation phase: Specimens were incubated in artificial saliva (pH 7.0) during the remaining 21 hours of incubation period including overnight incubation at 37°C.

Artificial saliva was made up with 1.5 mM calcium chloride, 0.9 mM sodium phosphate, 150 mM potassium chloride and 20 mM HEPES buffer and adjusted to pH 7.0. The solutions were changed daily.

## Surface microhardness assessment in post-treatment:

After the 28-day treatment, end-of-treatment surface microhardness values were determined (VHNremin) through the same parameters as baseline. Notches were cut along the perimeter of past locations of measurement.

## Calculation of percentages surface microhardness recovery:

The percentage recovery of surface microhardness was computed as a percentage of surface microhardness recovery.

## Scanning electron microscopy analysis:

SEM analysis was done on representative samples (n=5 each group). The specimens were dehydrated using progressing ethanol solutions (50, 70, 90, 100%) and attached on aluminum stubs and sputter-coated with gold-palladium. A field-emission scanning electron microscope with the accelerating voltage of 15 kV was used to examine surface morphology. Qualitative evaluation of the surface features and the dentinal tubule occlusion were performed on the micrographs obtained at standardized magnifications (x1000, x3000, x5000).

## Energy-dispersive x-ray spectroscopy analysis:

The same specimens were analysed using EDX to identify the elemental composition in terms of the content of calcium (Ca) and phosphorus (P) and calculate the ratio of Ca/P. Three standard areas on each of the surfaces of the specimen were measured and average values were obtained.

## Statistical analysis:

Statistical Package of Social Sciences version 25.0 was used to analyse data. The Shapiro-Wilk test was used to test the normal distribution of data. All quantitative variables have been used to compute descriptive statistics such as means and standard deviations. Comparison was done using one-way analysis of variance (ANOVA) and then the post-hoc test of Tukey was used to compare the two groups. The comparison of the baseline, post-demineralisation and post-remineralisation microhardness values was conducted in groups with the help of a paired t-test. The statistical significance was fixed at p<0.05.

## Results:

Baseline surface microhardness values were comparable across all groups with no statistically significant differences (p=0.892), confirming successful randomization. Following artificial demineralisation, all specimens demonstrated a significant reduction in surface microhardness (p<0.001), with mean percentage reduction ranging from 42.6% to 44.8% across groups (Table 1 - see PDF). Post-treatment analysis revealed significant differences in microhardness recovery among groups (F=48.62, p<0.001). All treatment groups demonstrated significantly higher post-remineralisation microhardness values compared to the control (p<0.001). The BioHAP group exhibited the highest post-remineralisation microhardness (50.14±4.52 VHN), followed by CPP-ACP (45.98±3.86 VHN) and Enamelon (45.06±4.24 VHN). The percentage surface microhardness recovery (%SMHR) demonstrated significant differences among groups (Table 2 - see PDF). BioHAP exhibited the highest remineralisation potential with 68.42±7.83% recovery, which was significantly superior to CPP-ACP (54.26± 6.91%, p<0.001) and Enamelon (49.18±8.24%, p<0.001). The difference between CPP-ACP and Enamelon was not statistically significant (p=0.214). The control group showed minimal recovery (9.42±4.86%), significantly lower than all treatment groups (p< 0.001). EDX analysis revealed significant differences in elemental composition following treatment (Table 3 - see PDF). Calcium and phosphorus content increased significantly in all treatment groups compared to demineralised baseline and control specimens. The BioHAP group demonstrated the highest calcium content (28.64±2.18 wt%) and phosphorus content (14.82±1.26 wt%), resulting in a Ca/P ratio of 1.94>0.12, closest to stoichiometric hydroxyapatite (1.67). SEM analysis revealed distinct surface morphological differences among groups. Demineralised specimens exhibited open dentinal tubules with irregular, rough surfaces and evidence of mineral loss. The intertubular dentin appeared porous with exposed collagen fibrils in some areas. BioHAP-treated specimens demonstrated extensive tubular occlusion with a uniform mineral layer covering the dentin surface. The deposited material appeared as spherical nanoparticle aggregates integrated with the underlying dentin structure. Most dentinal tubules showed complete or near-complete occlusion, with a smooth, homogeneous surface appearance. CPP-ACP-treated specimens exhibited partial tubular occlusion with irregular mineral deposits. The surface demonstrated areas of mineral deposition interspersed with partially open tubules. The deposited material appeared as amorphous precipitates, consistent with calcium phosphate formation. Enamelon-treated specimens showed variable tubular occlusion with crystalline deposits on the surface. Some tubules demonstrated occlusion by crystalline material, while others remained partially patent. The surface presented a heterogeneous appearance with areas of mineral deposition and exposed dentin. Control specimens showed minimal changes from the demineralised state, with predominantly open dentinal tubules and limited surface mineral deposition. Only superficial, patchy mineral precipitates were observed, attributed to calcium and phosphate from artificial saliva.

## Discussion:

The current research presents all-inclusive evidence to reveal that there is a high variation in the potential of remineralisation of the BioHAP, CPP-ACP and Enamelon in artificially demineralised root dentin. The null hypothesis was not accepted because BioHAP proved to have much higher remineralisation efficacy than the other agents tested. The results add useful information to the emerging extent of knowledge on the non-invasive management technique of root surface lesions. The high functionality of BioHAP in this research corresponds to the theoretical benefits of the biomimetic hydroxyapatite in dental remineralisation. The nanoparticle formulation allows close interaction with the demineralised dentin surface, allowing epitaxial growth of the nanoparticle on the existing hydroxyapatite crystals [6]. Recent studies have revealed that nano-hydroxyapatite particles have the ability to travel into the subsurface demineralised region where the mineral deposition may occur in depths in excess of the superficial layer [7]. Microhardness recovery rate of 68.42 per cent through the BioHAP treatment is a clinically significant amount of remineralisation, which may be enough to prevent lesion onset and mechanical functionality. These results have been observed in other studies on enamel remineralisation where nano-hydroxyapatite was found to be as effective as or even more effective than fluoride-based therapy [8]. The current research applies the above observances to root dentin, which proves the applicability of BioHAP to various dental substrates. The BioHAP action mechanism is complex and comprises several processes, such as adsorption to the surface, crystal nucleation and incorporation with the existing mineral structures.

The zinc-substituted formulation of this study can be beneficial in more ways, including antimicrobial action and improved crystal- forming kinetics [9]. The results of SEM observations that indicated that tubular occlusion is extensive indicate that it has the potential to be used in the clinical management of dentin hypersensitivity, as sealing of patent tubules decreases the hydrodynamic stimulation of pulpal mechanoreceptors. The reported moderate remineralisation efficacy of CPP-ACP with 54.26% microhardness recovery is in line with the known mechanism of the compound that entails the super saturation of the local environment with calcium and phosphate ions. The casein phosphopeptides can be used as delivery vehicles, where the stabilisation of these ions in bioavailable form happens and subsequently they become precipitated at the tooth surface as the hydroxyapatite precursors [2]. It has been established that remineralising activities of CPP-ACP on enamel are real and the current results indicate similar advantages on root dentin. The reason behind the slightly lesser efficacy of CPP-ACP in comparison to BioHAP could be associated with the variation in the mechanisms of delivering minerals. Whereas CPP-ACP offers ions that need to be further crystallised to hydroxyapatite, BioHAP offers already crystallised mineral particles with a crystalline structure comparable to natural tooth mineral [1]. This inherent disparity might explain the faster and greater remineralisation that is seen with BioHAP treatment. Enamelon showed similar capability to remineralisation with 49.18% recovery of microhardness due to CPP-ACP. The integration of stannous fluoride and calcium sodium phosphosilicate bioactive glass is a new solution on the basis of the synergistic effects [16]. The bioactive Glass constituent releases calcium, phosphate and sodium ions when in contact with aqueous media; whereas stannous fluoride gives the bioactive glass its acid resistance and antimicrobial properties. The similarity in the performance of Enamelon and CPP-ACP indicates that not all remineralising technologies can bring the same results via the same means. Nevertheless, the particular benefits of each system, such as antimicrobial efficacies, patient acceptability and cost-effectiveness, might have a role in clinical treatment choice other than remineralisation efficacy itself [3]. The EDX analysis showed marked gains in the contents of calcium and phosphorus after all active agents were used, which confirmed the fact of mineral deposition and not just hardening on the surface. The Ca/P ratios recorded were close to stoichiometric hydroxyapatite, implying that crystalline mineral phases were formed and not amorphous precipitates [4]. The best restoration of the microhardness in BioHAP-treated specimens is associated with the maximum calcium and phosphorus level of this group. SEM results are morphological evidence in support of the quantitative data. The massive tubular occlusion seen in BioHAP therapy is indicative of two-fold benefits of the treatment with clinical use, which are remineralisation of demineralised dentin and cure of dentin hypersensitivity. The modern knowledge acknowledges the significance of dental tubule patency in the aetiology of hypersensitivity and tubular occlusion is one of the major therapeutic focuses [5].

The pH cycling model used in this study constitutes strength in the design of an experiment since it models the dynamic oral environment better than the remineralisation protocols that remain static. The conditions in the daily pH cycling of acids simulate those of the dietary exposure to acid and the introduction of intermittent treatment application compares with oral hygiene practices [17]. This approach will improve the clinical pertinence of results as opposed to continuous immersion procedures. The artificial demineralisation regimen effectively produced standardised early carious lesions (~44% surface microhardness loss), suitable for evaluating remineralisation potential via non-invasive approaches [18]. Even more severely demineralised samples may exhibit alternative response patterns, warranting future investigation. Key limitations temper interpretation: this *in vitro* design cannot replicate the oral environment's complexity, including salivary proteins, pellicle formation, and biofilm dynamics. While the 28-day treatment phase adequately demonstrated remineralisation potential, it may not reflect long-term mineral deposit stability. [19]. These results need to be verified with randomised controlled trials investigating the effects of these interventions on outcomes related to patients, such as caries arrest, hypersensitivity reduction and lesion reversal. Also, patient compliance, product acceptability and cost- effectiveness are other factors to be considered in treatment recommendations [20]. Future directions involve longer pH cycle studies to determine remineralisation stability, combination therapy studies that would help in determining the synergistic effects of various remineralising agents and clinical studies to ascertain the *in vitro*findings in the patient group that is highly susceptible to root caries [3]. The results of the presented research prove the prospect of BioHAP as the first-line agent of remineralisation in the case of root dentin lesion and the relevance of CPP-ACP and Enamelon as the alternatives remains. The choice of these agents in clinical practice can be based on some factors related to the individual patients and these factors can include the history of fluoride exposure, the dietary habits and the patients 'choices between the fluoride-containing products and the fluoride-free products.

## Conclusion:

BioHAP demonstrated the highest remineralisation efficacy on root dentin, with significantly greater microhardness recovery, mineral content and dentinal tubule occlusion compared to CPP-ACP and Enamelon. SEM and EDX analyses confirmed BioHAP's superior formation of a homogenous mineral layer and calcium-phosphorus ratios approximating stoichiometric hydroxyapatite. These findings suggest BioHAP as a promising fluoride-free alternative for managing root caries and dentin hypersensitivity, warranting clinical validation through randomised controlled trials.
